# Ultrasound-mediated paclitaxel-loaded EGFR nanoparticles for targeted therapy in breast cancer

**DOI:** 10.1039/d5ra01016k

**Published:** 2025-07-04

**Authors:** Zhenbin Xu, Hongpeng Duan, Yuling Shi, Zixia Zhou, Zhuo Wei, Xuechen Qian, Jian Lu, Yuemingming Jiang, Feng Mao, Nianyu Xue, Shengmin Zhang

**Affiliations:** a Department of Ultrasonography, The First Affiliated Hospital of Ningbo University China fyyzhangshengmin@nbu.edu.cn; b Health Science Center, Ningbo University Ningbo China

## Abstract

Paclitaxel (PTX)-loaded nanoparticles based on poly(lactic-*co*-glycolic acid) (PLGA) represent a promising platform for improving chemotherapeutic efficacy in triple-negative breast cancer (TNBC), a highly aggressive subtype with limited therapeutic targets and poor clinical outcomes. To address challenges of nonspecific distribution and systemic toxicity associated with conventional PTX treatment, we designed a multifunctional nanocarrier system integrating active targeting and ultrasound responsiveness. The nanoparticles (PTX@TNPs) were prepared using a double emulsion method, encapsulating PTX and perfluoropentane (PFP) in a PLGA matrix, followed by surface conjugation of GE11 peptides targeting the epidermal growth factor receptor (EGFR) *via* EDC/NHS chemistry. Comprehensive physicochemical characterization revealed favorable particle size, colloidal stability, and drug loading efficiency. *In vitro* studies using EGFR-overexpressing MDA-MB-231 cells demonstrated significantly enhanced cellular uptake and cytotoxicity of PTX@TNPs, especially under ultrasound irradiation. *In vivo*, PTX@TNPs combined with ultrasound markedly inhibited tumor growth, suppressed microvessel density, and induced apoptosis in a TNBC xenograft model, while exhibiting reduced systemic toxicity compared to free PTX. Histological and immunohistochemical staining confirmed downregulation of proliferation marker Ki-67 and angiogenesis marker CD31 in tumor tissues following treatment. These findings highlight the synergistic therapeutic potential of combining EGFR-mediated active targeting with ultrasound-triggered drug release and underscore the translational value of PTX@TNPs as a safe and efficient nanoplatform for TNBC treatment.

## Abstract 2

Schematic diagram of preparation of drug-loaded nanoparticles targeting EGFR and killing breast cancer in mice under the ultrasound trigger.

## Graphical abstract

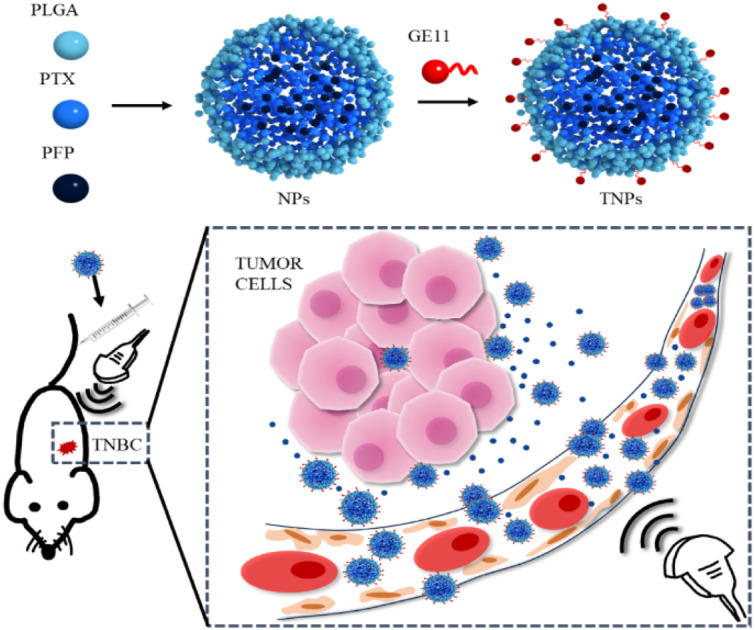

## 1 Introduction

Breast cancer is the most common malignant tumor in women, recently surpassing lung cancer as the most prevalent cancer worldwide.^1^ Breast cancer is characterized by significant molecular heterogeneity, which influences treatment decisions based on distinct molecular subtypes.^2–6^ Among its subtypes, triple-negative breast cancer (TNBC) is defined by the absence of estrogen (ER), progesterone (PR), and HER-2 receptors.^7^ TNBC accounts for approximately 10–20% of breast cancer cases and presents a significant therapeutic challenge, with a 5-year survival rate of about 65% and poor prognosis.^8–11^ Early intervention is therefore critical. While surgery remains a primary treatment, chemotherapy is one of the few available systemic options. Paclitaxel (PTX) is a key chemotherapeutic agent that stabilizes microtubules, disrupts mitosis, and demonstrates potent anti-tumor activity.^12–14^ However, high doses of PTX can harm healthy tissues, causing systemic toxicity and adverse effects such as anemia, nausea, vomiting, and alopecia.^15^ These side effects limit its therapeutic efficacy, prompting researchers to explore strategies to enhance drug delivery to target tissues while minimizing systemic toxicity.

Nano-drug delivery systems have emerged as a promising approach in cancer therapy, improving drug efficacy by increasing targeted accumulation and reducing systemic side effects.^16^ Nano-drug carriers offer advantages such as enhanced solubility, controlled release, and biocompatibility. The enhanced permeability and retention (EPR) effect enables macromolecules like liposomes and nanoparticles to penetrate tumor tissues *via* leaky tumor vasculature, allowing prolonged retention and increased local drug concentration.^17^ Among nanoparticle materials, PLGA, an FDA-approved biodegradable polymer, has gained attention due to its rapid metabolism into lactic and glycolic acids, which exhibit low systemic toxicity.^18^ However, the passive targeting capability of nanoparticles *via* the EPR effect remains limited.^19–21^

To overcome these limitations, researchers have focused on modifying nanoparticle surfaces to enhance receptor-mediated endocytosis and improve active targeting.^22–24^ Epidermal growth factor receptor (EGFR), overexpressed in up to 80% of TNBC cells,^7^ represents a promising target for therapy. In this study, we utilized the polypeptide GE11 (YHWYGYTPQNVI), initially identified through phage display technology, which specifically binds EGFR with high affinity. The GE11 peptide used in this study was originally identified *via* phage display screening and demonstrated a dissociation constant (*K*_d_) of approximately 22 nM for EGFR, as reported by Li *et al.*,^25^ indicating a high binding affinity suitable for targeted delivery applications.

Although the EPR effect is a cornerstone of nano-drug delivery, its variability between animal models and human applications poses challenges for clinical translation.^26^ For tumors with low EPR effects, ultrasound-targeted microbubble destruction (UTMD) offers a viable method to enhance drug delivery.^27–29^ UTMD temporarily increases tissue permeability by inducing microvascular and cell membrane pores, thus facilitating drug penetration.^30^ This technique has been successfully integrated into various therapeutic modalities, including chemotherapy, gene therapy, and sonodynamic therapy.^31^ Moreover, UTMD can induce microbubble oscillation and collapse, releasing therapeutic agents directly into targeted tissues.^32^

**Fig. 1:**
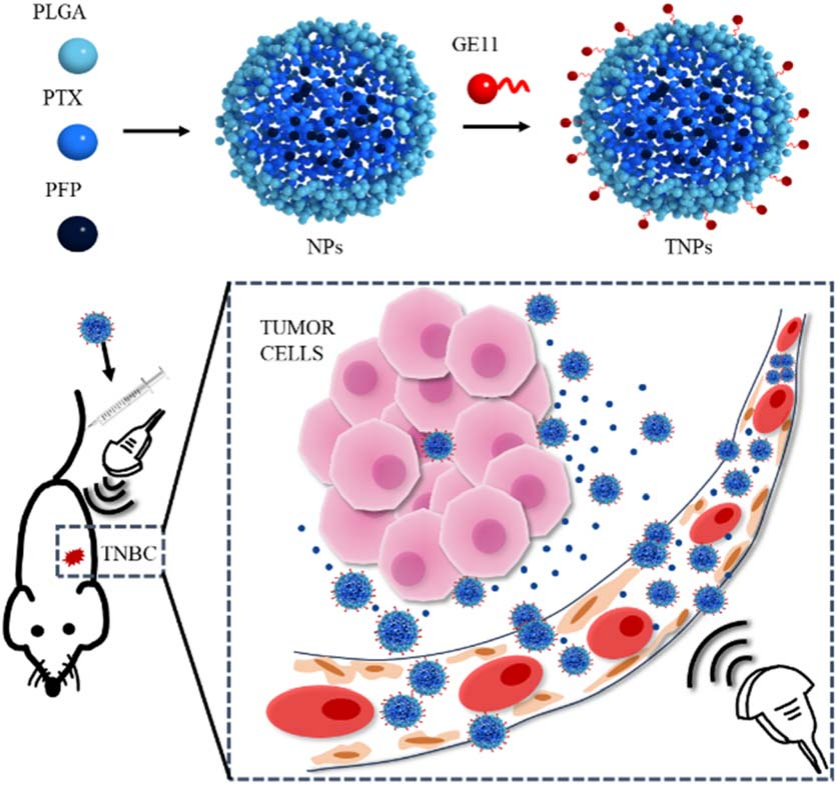
Schematic diagram of preparation of drug-loaded nanoparticles targeting EGFR and killing breast cancer in mice under the ultrasound trigger.

In this study, we developed a novel nano-delivery system (PTX@TNPs) that combines targeted and ultrasound-responsive drug release capabilities. This system employs PLGA as the shell, PFP droplets and PTX as the core, and GE11-functionalized nanoparticles targeting EGFR. Designed specifically for TNBC treatment, this carrier enables precise and efficient PTX delivery at the tumor site, enhancing therapeutic efficacy while minimizing systemic side effects (Fig. 1).

## 2 Materials and methods

### 2.1 Cell lines, animals, and materials

The MDA-MB-231 breast cancer cell lines were obtained from the Chinese Academy of Sciences Cell Bank (Shanghai, China). Female Balb/c nude mice were purchased from Zhejiang Vital River Laboratory Animal Technology Co., Ltd (Jiaxing, China). PLGA (50/50, MW 2 kDa) was obtained from Yare Biotech, Inc. (Shanghai, China). Paclitaxel (PTX), polyvinyl alcohol (PVA), 1-ethyl-3-(3-dimethylaminopropyl) carbodiimide hydrochloride (EDAC), and *N*-hydroxysuccinimide (NHS) were sourced from Aladdin Industrial Inc. (Shanghai, China). Dichloromethane (DCM) and HPLC-grade methanol were procured from Sinopharm Chemical Reagent Co., Ltd (Shanghai, China). Coumarin 6 (C6) was purchased from Solarbio Science & Technology Co., Ltd (Beijing, China). Perfluoro-*n*-pentane (C5F12, PFP) was supplied by Toronto Research Chemicals (Toronto, Canada). GE11 (Tyr-His-Trp-Tyr-Gly-Tyr-Thr-Pro-Gln-Asn-Val-Ile-NH2) was synthesized by Nanjing Peptide Biotech Ltd (Nanjing, China). The TUNEL kit and hematoxylin and eosin (H & E) staining kit were acquired from Beyotime Biotech Co., Ltd (Hangzhou, China). The Annexin V-FITC/PI Apoptosis Detection Kit was purchased from BD Biosciences (San Diego, USA). Antibodies against Ki-67 and CD31 were obtained from Cell Signaling Technology (Beverly, USA).

### 2.2 EGFR expression analysis in breast cancer

To analyze EGFR expression in breast cancer, The Cancer Cell Line Encyclopedia (CCLE) online databases were utilized. CCLE database was used to examine EGFR expression in various breast cancer cell lines, with a particular focus on comparing TNBC cell lines with those from other breast cancer subtypes.

Protein samples were extracted from MCF-10A, MDA-MB-231, MCF-7 and SKBR-3 cells using RIPA lysis buffer supplemented with protease inhibitor cocktail. Lysates were centrifuged at 12 000×*g* for 15 min at 4 °C, and supernatants were quantified *via* BCA assay. Equal amounts of protein were separated by 10% SDS-PAGE and transferred onto PVDF membranes using a semi-dry transfer system.

Membranes were blocked with 5% non-fat milk in TBST for 1 h at room temperature, then incubated overnight at 4 °C with antibodies: anti-GAPDH (1 : 2000, CST) and anti-EGFR (1 : 1000, CST). After TBST washes (5 × 10 min), membranes were probed with HRP-conjugated secondary antibodies (1 : 20 000, MCE) for 1 h at RT. Protein bands were visualized using ECL substrate and imaged with a ChemiDoc™ MP system (Bio-Rad).

### 2.3 Preparation of PTX@NPs

The preparation of non-targeted nanoparticles (NPs) was conducted following a previously described method.^33^ Non-targeted PTX-loaded nanoparticles (PTX@NPs) were prepared by a double emulsion method6. Briefly, 100 mg of PLGA and 4 mg of PTX were dissolved in 4 mL of dichloromethane. This solution was placed in a 50 mL centrifuge tube, and 400 μL of perfluoropentane droplets were added. The first emulsion was created using an ultrasonic cell pulverizer in an ice bath. The emulsion was then quickly transferred to 20 mL of an ice-cold PVA solution for a second emulsion. The mixture was stirred magnetically for 4 hours, followed by low-temperature centrifugation. The resulting precipitate was resuspended in 5 mL of deionized water, and the suspension was freeze-dried to yield powdered PTX@NPs.

### 2.4 Preparation of PTX@TNPs

Peptide ligand was conjugated *via* EDC/NHS chemistry.^34^ To prepare targeted nanoparticles (PTX@TNPs), 20 mg of PTX@NPs was dissolved in 20 mL of PBS in a beaker. Then, 0.6 mL of EDC solution and 0.4 mL of NHS solution were added, and the mixture was stirred magnetically. After 20 minutes, 5 mL of GE11 solution was introduced, and the reaction continued for 16 hours. The resulting solution was centrifuged (12 500 g min^−1^, 45 minutes, 4 °C), and the precipitate was freeze-dried to obtain PTX@TNPs.

### 2.5 Characterization

The particle size and zeta potential of the nanoparticles were measured by Dynamic Light Scattering (DLS) using a Zetasizer Nano ZS90 instrument (Malvern, Worcestershire, England). The morphology of PTX@NPs and PTX@TNPs was observed using Transmission Electron Microscopy (TEM, TF20, FEI, USA). The composition of the TNPs was analyzed using Differential Scanning Calorimetry (DSC, Mettler Toledo, Switzerland) and Fourier Transform Infrared Spectroscopy (FT-IR, PerkinElmer, USA). The light absorption properties of nanoparticles were measured using ultraviolet-visible spectrophotometry (UV-3600i Plus, Japan). PTX@TNPs nanoparticles were stored at 4 °C. Particle size was measured using a Malvern Zetasizer on days 1, 3, 5 and 7 to evaluate nanoparticle stability. PTX@TNPs were ultrasonically irradiated *in vitro*, and particle size and morphology were examined by Malvern Zetasizer and inverted fluorescence microscopy to verify the ultrasonic responsiveness of PFP.

### 2.6 Drug loading and encapsulation efficiency of PTX@TNPs

The encapsulation and loading efficiencies of PTX@TNPs were determined by High-Performance Liquid Chromatography (HPLC, Agilent 1260, Agilent Technologies, Santa Clara, CA, USA). The mobile phase consisted of a 70 : 30 methanol–water mixture, with a detection wavelength of 227 nm. Encapsulation efficiency was calculated as the amount of PTX encapsulated in NPs divided by the total amount of PTX added, multiplied by 100%. Loading efficiency was determined as the amount of PTX encapsulated in NPs divided by the total amount of PLGA polymer used for NP preparation, multiplied by 100%.

### 2.7 *In vitro* drug release assay

PTX@TNPs nanoparticles were placed in PBS (pH 6.5) containing methyl alcohol as the release medium and incubated in a thermostatic shaker at 37 °C and 100 rpm. At predetermined time points (2 h, 4 h, 12 h, 18 h, 24 h, 36 h, and 48 h), 1 mL of the release medium was withdrawn to measure the absorbance at 227 nm, and an equal volume of fresh PBS was added to maintain a constant volume. To assess drug release under ultrasound conditions, the nanoparticles were also tested in the presence or absence of ultrasound (1 MHz, 0.4 W cm^−2^, 5 minutes). The drug release concentration of PTX@TNPs were determined by HPLC.

### 2.8 Hemolysis test

Blood from healthy Balb/c mice was collected in EDTA-treated tubes and centrifuged with PBS solution. The supernatant was discarded, and the remaining solution was clarified. Resuspended red blood cells were then incubated with various concentrations of NPs or TNPs in PBS. After centrifugation, the absorbance at 577 nm was measured using an enzyme-labeling instrument. The hemolysis rate was calculated using the formula: hemolysis rate (%) = (OD_sample_ − OD_negative_)/(OD_positive_ − OD_negative_) × 100%. A hemolysis rate greater than 5% was considered indicative of hemolysis.

### 2.9 *In vitro* cellular uptake

#### 2.9.1 Quantitative study

To assess cellular uptake, Coumarin 6 was used in place of PTX to prepare Coumarin-6@NPs and Coumarin-6@TNPs. The nanoparticles' uptake by MDA-MB-231 cells was observed using a Confocal Laser Scanning Microscope (CLSM). The cells were seeded in 12-well plates and incubated with nanoparticles for 2 and 4 hours. After washing with PBS, the cells were fixed with 4% paraformaldehyde, stained with DAPI, and imaged in five random visual fields.

#### 2.9.2 Qualitative study

Cell uptake was quantitatively assessed by flow cytometry. MDA-MB-231 cells were seeded into 6-well plates, incubated with Coumarin-6@NPs and Coumarin-6@TNPs for 2 and 4 hours, and then processed for flow cytometric analysis.

### 2.10 *In vitro* cytotoxicity

Cell viability was evaluated using the CCK-8 assay. The effect of blank nanoparticles on cell viability was initially tested, followed by the cytotoxicity of PTX-loaded nanoparticles. Cells were treated with various concentrations of NPs and TNPs (0–1000 μg mL^−1^) and incubated for 48 hours, after which cell viability was assessed by measuring absorbance at 450 nm. The inhibition rate was calculated as: Inhibition rate (%) = (1 − OD_study group_/OD_blank group_) × 100%. The cytotoxicity of drug-loaded nanoparticles was further evaluated in several experimental groups, including control, free PTX, PTX@NPs, PTX@TNPs, and PTX@TNPs + US.

### 2.11 Apoptosis assays

The MDA-MB-231 cells were seeded in 6-well plates and treated with the following groups: control, free PTX, PTX@NPs, PTX@TNPs, and PTX@TNPs + US for 24 h. After the addition Annexin V-FITC binding buffer, the apoptotic cells were stained with Annexin V-FITC (MCE) and PI(MCE) for 20 min. Apoptotic cells was detected by flow cytometry. Apoptosis was also evaluated using live/dead cell staining. Tumor cells were seeded into 6-well plates, incubated overnight, and then co-cultured with PBS, free PTX, PTX@NPs, PTX@TNPs, and PTX@TNPs + US for 24 h. The cells were stained with calcein-AM (Elabscience) and PI (Elabscience) for 30 min and observed using an inverted fluorescence microscope.

### 2.12 Animal models and groups

Female Balb/c nude mice (18–20 g, 4–6 weeks old) were used for *in vivo* studies. The mice were implanted with MDA-MB-231 cells to establish a breast cancer tumor model. Tumor size was measured using a caliper, and tumors with volumes between 40–60 mm^3^ were selected for further studies.

The mice were randomly divided into six treatment groups, each with five mice: control (saline), US, free PTX, PTX@NPs, PTX@TNPs, and PTX@TNPs + US. Tumor treatments were administered every three days for a total of five injections, with the final treatments followed by euthanasia on day six. The ultrasound conditions were: probe frequency of 1.0 MHz, sound intensity of 0.4 W cm^−2^, duty cycle of 50%, and irradiation time of 5 minutes.

### 2.13 *In vivo* therapeutic effect

The therapeutic efficacy of each treatment was assessed by monitoring tumor volume, tumor weight, and body weight. Tumor volume was calculated using the formula: *v* = (*L* × *W*^2^)/2, where *L* is the longest diameter and *W* is the shortest diameter of the tumor. The relative tumor ratio was determined as *v*/*v*_0_, with *v* representing the immediate tumor volume and *v*_0_ representing the primary tumor volume. After recording the measurements, the mice were euthanized, and the tumor tissue was extracted and weighed. The tumor samples were then fixed with paraformaldehyde, embedded in paraffin, and sliced using a paraffin slicer.

The superb microvascular imaging (SMI) of breast metastases in mice was assessed using a Mindray ultrasound machine before each treatment, as well as 3 and 6 days after the last treatment. The study procedure involved anesthetizing the mice, applying a coupling agent to the subcutaneously implanted tumor, and covering it with an ultrasonic coupling pad. The linear probe L15-3 was selected for performing SMI three times. The tumor Vascular Index (VI) changes were quantitatively analyzed and plotted.

The harvested organs were dissected and subjected to histological examination using hematoxylin and eosin (H & E) staining. Immunohistochemical staining of tumors was carried out to detect the nuclear antigen Ki-67. The TdT-mediated dUTP nick-end labeling technique (TUNEL) staining was also performed. Furthermore, the expression of microvessels in the tumor tissues was performed using CD31 staining.

### 2.14 Toxicity evaluation *in vivo*

Six groups of healthy 6-week-old Balb/c mice were randomly assigned and treated with different methods. Three days after the final treatment, the mice were euthanized, and blood samples and essential organs (liver, spleen, heart, lung, kidney) were collected for analysis. Blood indicators such as ALT, AST, CREA, and BUN were measured, and HE staining was used to observe any morphological changes in vital organs.

### 2.15 Statistical analysis

All data were analyzed using GraphPad Prism 9.0 software. Results are expressed as mean ± standard deviation (SD). One-way ANOVA followed by Tukey's multiple comparison test was used for group comparisons. Statistical significance was defined as *p* < 0.05.

## 3 Results and disscussion

### 3.1 EGFR expression in breast cancer

**Fig. 2:**
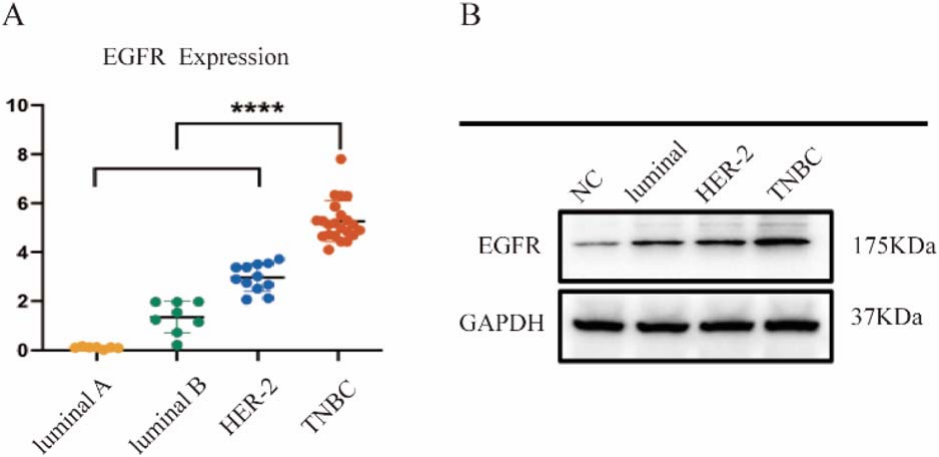
EGFR expression in breast cancer. (A) Bioinformatics analysis of EGFR levels in tumor tissues of various subtypes of breast cancer (CCLE). (B) Western blot of EGFR in MCF-10A (normal cells), MDA-MB-231 (TNBC), MCF-7 (Luminal A), and SK-BR-3 (HER2+) cells.

The expression levels of EGFR show significant differences across various breast cancer subtypes, which may be closely associated with clinical prognosis. Analysis of the CCLE database revealed that EGFR expression in breast cancer tumor tissues is significantly higher than in other tissues (Fig. 2A). This result suggests that the role of EGFR in breast cancer progression may differ from that in other cancer types. As shown in Fig. 2B, the expression level of EGFR (∼175 kDa) was markedly increased in MDA-MB-231 cells *versus* MCF-10A, MCF-7 and SKBR-3 cells.

### 3.2 Characterization of PTX@NPs and PTX@TNPs

**Fig. 3:**
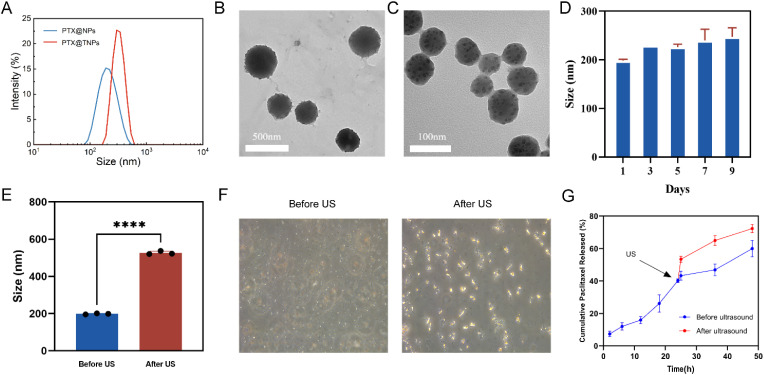
Characterization of PTX@TNPs. (A) Size distribution of PTX@NPs and PTX@TNPs measured by DLS. (B and C) TEM images of PTX@TNPs (B) and PTX@NPs (C). (D) Particle size changes of PTX@TNPs during 9-day storage in PBS (pH 7.4). (E) Hydrodynamic size of PTX@TNPs before and after ultrasound (US) exposure (****p < 0.0001). (F) Optical images of PTX@TNPs before and after US treatment. (G) Cumulative paclitaxel release from PTX@TNPs with or without US stimulation.

The particle size of PTX@NPs was measured to be 140.0 ± 1.3 nm (PDI = 0.09 ± 0.02, *n* = 3), with a zeta potential of −17.2 ± 0.4 mV. In contrast, the average particle size of PTX@TNPs was 198.4 ± 2.6 nm (PDI = 0.25 ± 0.003, *n* = 3), with a zeta potential of −13.3 ± 0.4 mV (Fig. 3A), suggest that the nanoparticles are suitable for long circulation times and efficient tumor accumulation *via* the enhanced permeability and retention (EPR) effect. The EPR effect allows nanoparticles to exploit the leaky vasculature of solid tumors for improved drug delivery.^35,36^ The encapsulation efficiency (EE%) of PTX in PTX@TNPs was found to be 40.0 ± 0.6% (*n* = 3). Transmission electron microscopy (TEM) analysis confirmed that both PTX@NPs and PTX@TNPs exhibited spherical core–shell structures (Fig. 3B and C). Additionally, the colloidal stability of the nanomedicine was evaluated by monitoring the size of the NPs in PBS solutions at different pH values. The size remained relatively stable across in a solution with a pH of 7.4, indicating that the nanomedicine exhibited good colloidal stability (Fig. 3D). After ultrasonic irradiation, the particle size of PTX@TNPS increased from 100 nm to 500 nm, and the particle size increased obviously under microscope (Fig. 3E and F). In this study, PTX@TNPs demonstrated a sustainable drug release behavior up to 48 h. Additionally, our results showed that PTX@TNPs exhibited a dose-dependent drug release behavior in response to ultrasound with a cumulative PTX release reaching 72.3 ± 2.5% at 48 h (Fig. 3G).

**Fig. 4:**
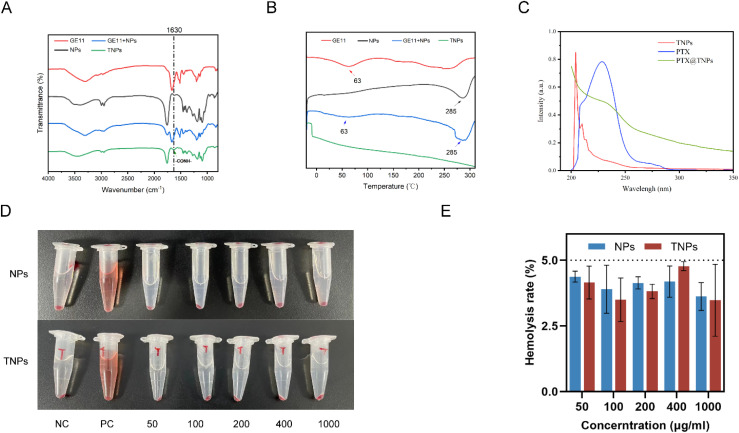
(A) FT-IR spectrums (B) DSC images (C) UV-vis spectrophotometry. (D and E) The hemolysis rates of different Nps and TNps group concentrations were less than 5%. Negative control group, positive control group, 50 μg mL^−1^ NPs/TNPs, 100 μg mL^−1^ NPs/TNPs, 200 μg mL^−1^ NPs/TNPs, 400 μg mL^−1^ NPs/TNPs, and 1000 μg mL^−1^ NPs/TNPs from left to right.

TNPs were successfully synthesized *via* amide bond formation between the carboxyl group of PLGA and the amino group of GE11 using EDC/NHS coupling chemistry. The structure of the TNP conjugate was further confirmed by Fourier transform infrared (FTIR) spectroscopy. Compared to the NP spectrum, TNPs exhibited a sharper and more prominent peak at 1630 cm^−1^, corresponding to the –CO–NH– stretching vibration, which is indicative of amide bond formation between PLGA and GE11 (Fig. 4A). Differential scanning calorimetry (DSC) analysis of the TNP conjugate revealed exothermic peaks for GE11 and NPs at 63 °C and 285 °C, respectively. When the two components were mixed, exothermic peaks were observed at both temperatures (Fig. 4B). Importantly TNPs did not show any exothermic peaks at these temperatures, indicating the successful synthesis of the TNPs. Furthermore, the UV-vis absorbance plot shows PTX@TNPs do not have strong absorbance at 227 nm compare to PTX (Fig. 4C).

### 3.3 Hemolysis test

The supernatants of both the NPs and TNPs groups exhibited no distinct red color at any of the five tested concentrations, as observed with the naked eye. The hemolysis rate for both groups was below 5% (*n* = 3), indicating that the two nanoparticles had minimal impact on red blood cells (Fig. 4D and E).

### 3.4 *In vitro* cellular uptake

**Fig. 5:**
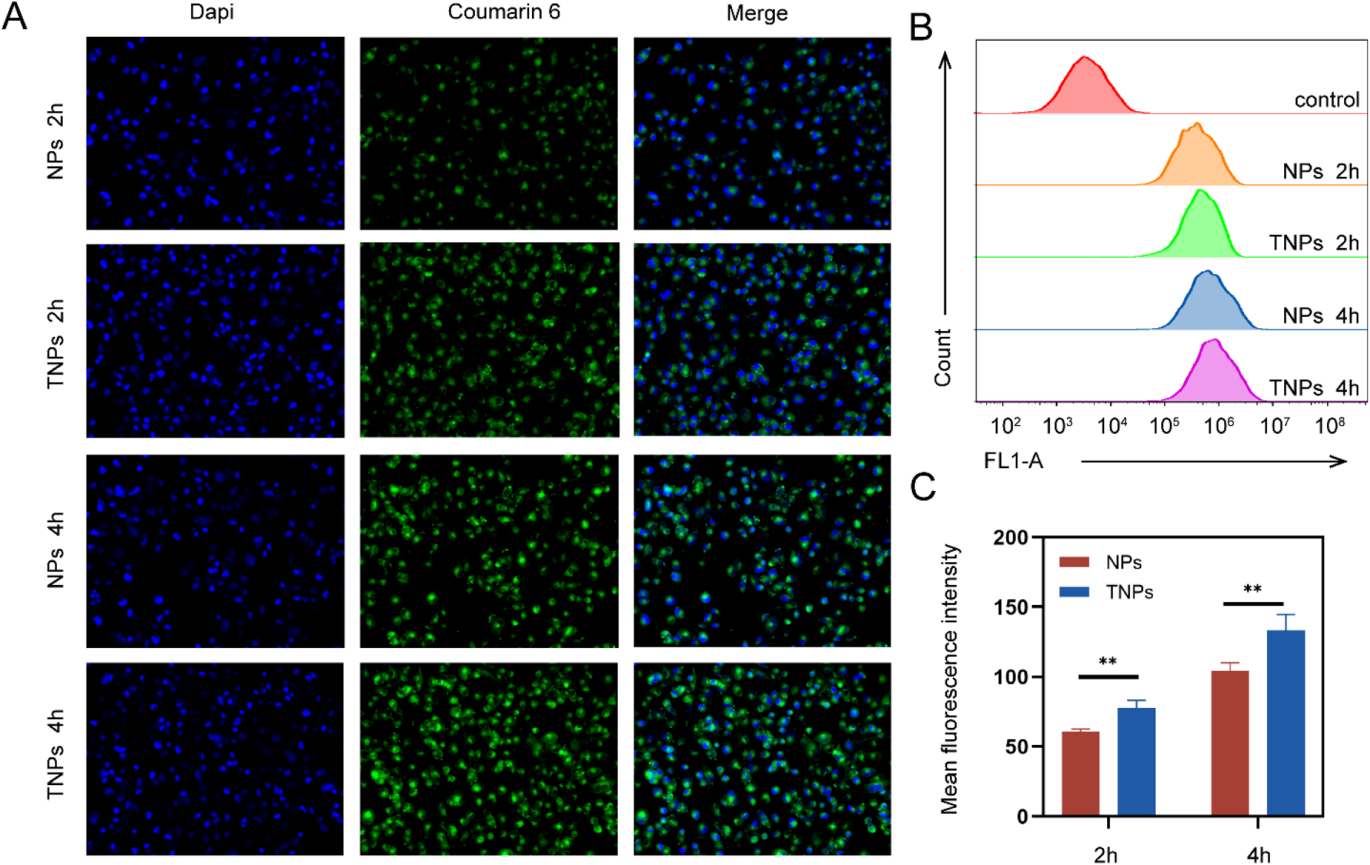
Cellular uptake of TNPs. (A) CLSM images of MDA-MB-231 incubated with Coumarin 6@NPs and Coumarin 6@TNPs *in vitro* for two hour, and four hour(scale bar: 50 μm); (B and C) flow cytometry analysis was used to analyze the difference of MFI (mean ± SD), **p < 0.01.

A confocal laser scanning microscope was used to assess the cellular uptake and distribution of Coumarin-6@NPs and Coumarin-6@TNPs. The nuclei were stained with DAPI, which emits blue fluorescence. As shown in Fig. 5, after 2 and 4 hours of incubation, the green fluorescence of Coumarin-6 was detected within the nuclei of MDA-MB-231 cells treated with either Coumarin-6@NPs or Coumarin-6@TNPs. The uptake of both NPs and TNPs by MDA-MB-231 cells increased gradually with a longer incubation period (from 2 hours to 4 hours). Notably, the uptake of TNPs was more pronounced, with more intense green fluorescence observed after 4 hours of incubation. To objectively compare the uptake of the two nanoparticles, flow cytometry analysis revealed a significant increase in the uptake of TNPs by MDA-MB-231 cells after 4 hours. Additionally, the mean fluorescence intensity (MFI) of Coumarin-6 uptake by the nanoparticles was quantitatively analyzed, showing a significant difference between specifically targeted and non-targeted nanoparticles at both 2 hours (*P* = 0.0012, *n* = 4) and 4 hours (*P* = 0.0039, *n* = 4). This targeting mechanism significantly enhances the cellular uptake of PTX in TNBC cells, ensuring the drug reaches the tumor site more effectively.

### 3.5 *In vitro* anti-tumor efficacy of the PTX@TNPs

**Fig. 6:**
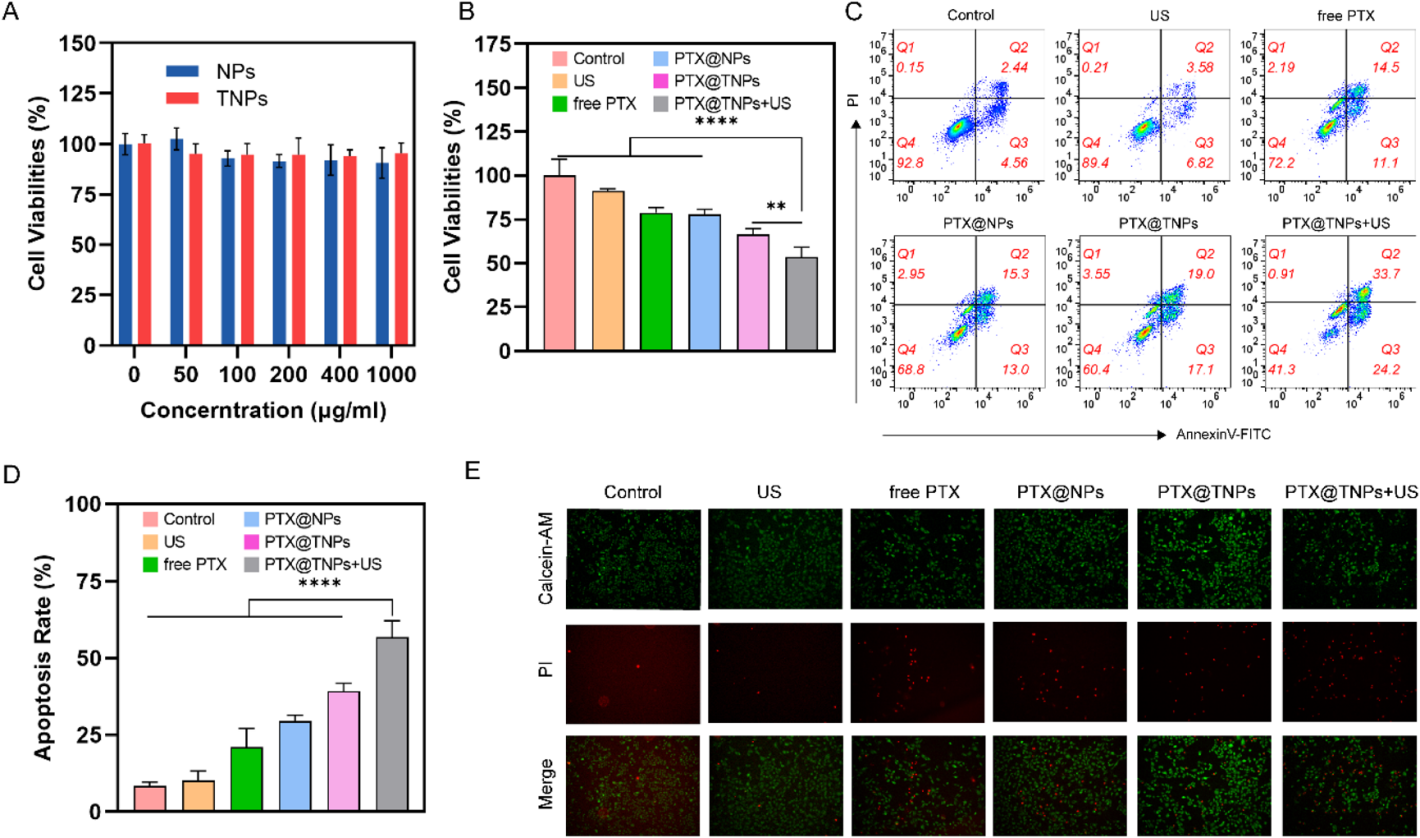
*In vitro* anti-tumor efficacy of the PTX@TNPs. (A) The NPs and TNPs groups showed no apparent cytotoxicity, and the cell viability rate was above 90%. (B) Showed a significant difference between the PTX@TNPs + US group and the other groups(**p < 0.01, ****p < 0.0001). (C and D) Flow cytometric analysis of apoptosis using Annexin V-FITC/PI, confirming that PTX@TNPs + US induces the highest rate of apoptosis in MDA-MB-231 cells (E) Calcein-AM/PI live-dead co-staining, which visually distinguishes viable and apoptotic cells.

The effect of NPs and TNPs on the viability of the human breast cancer MDA-MB-231 cell line was evaluated after a 24-hour incubation period. The results demonstrated that cell viability remained above 90.6% for NPs and 93.9% for TNPs, even at concentrations ranging from 0.05 to 1.0 mg mL^−1^. This suggests that both NPs and TNPs are biocompatible and cause minimal harm to MDA-MB-231 cells (Fig. 6A).

Nanoparticle-based drug delivery systems have shown considerable promise in oncology, particularly with antibodies or peptides to improve targeting efficiency.^37,38^ This study demonstrates that the use of GE11 peptides enhances the accumulation of PTX in EGFR-expressing TNBC cells. Further analysis revealed differences in the viability of MDA-MB-231 cells across six experimental groups. The cell survival rate in the ultrasound (US) group was 91.2% ± 1.3%, the free PTX group was 78.7% ± 3.0%, the PTX@NPs group was 77.9% ± 2.8%, the PTX@TNPs group was 66.3% ± 3.4%, and the PTX@TNPs + US group was 53.5% ± 5.7%. The PTX@TNPs + US group exhibited the most potent toxic effect on breast cancer cells. As shown in Fig. 6B, there was a significant difference between the ultrasound group and the PTX@TNPs group (*P* = 0.0058, *n* = 5), as well as significant differences when compared with the other groups (*P* < 0.0001, *n* = 5). These results indicate that the toxicity of nanoparticles increases after surface modification. Moreover, nanoparticles demonstrated an ultrasonic response, significantly enhancing the cytotoxic effect of ultrasound on cells.

The apoptosis rates of MDA-MB-231 cells treated with PTX@TNPs with ultrasound was 56.9%, respectively (Fig. 6C), with statistically significant difference compared to that induced by the other groups (Fig. 6D). In line with this, live/dead staining was used to evaluate the toxic effects of various PTX formulations on MDA-MB-231 (Fig. 6E). Almost no dead cells (red color) were detected in the control group and ultrasound group, whereas a large number of dead cells were observed after treatment with PTX-containing drugs including free PTX, PTX@NPs, PTX@TNPs, and PTX@TNPs with ultrasound.

These findings suggest that surface modification of nanoparticles can enhance their therapeutic potential, particularly when combined with ultrasound treatment and ultrasound irradiation could trigger the release of drugs from nanocarriers by inducing a liquid-to-gas transition in perfluorocarbon (PFP) encapsulated within the nanoparticles, leading to nanoparticle rupture and localized drug release similar to previous research.^39,40^

### 3.6 Toxicity evaluation *in vivo*

**Fig. 7:**
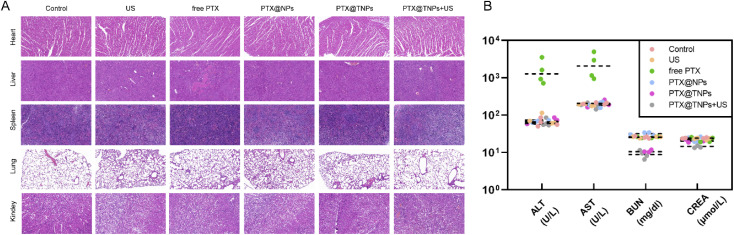
(A) H & E staining and histological analyses of organs (scale bar: 100 μm) and (B) AST, ALT, CRE, and BUN levels in Balb/c mice treated with PBS, ultrasonic cavitation, free PTX solution, PTX@NBs, PTX@TNBs or ultrasonic cavitation combined PTX@TNB were quantified.

As shown in Fig. 7, the free PTX group had a significant impact on mice, as evidenced by elevated levels of liver enzymes ALT and AST in the blood biochemistry, as well as liver histological damage observed through, HE staining. In contrast, the PTX@NPs, PTX@TNPs, and PTX@TNPs + US groups exhibited minimal signs of these adverse effects, indicating that the nanoparticle formulations effectively mitigated liver injury.

### 3.7 *In vivo* therapeutic effect

During the treatment period, no significant differences in body weight were observed among the groups, indicating that the mice remained in good condition without signs of cachexia or chemotherapy-related side effects.

**Fig. 8:**
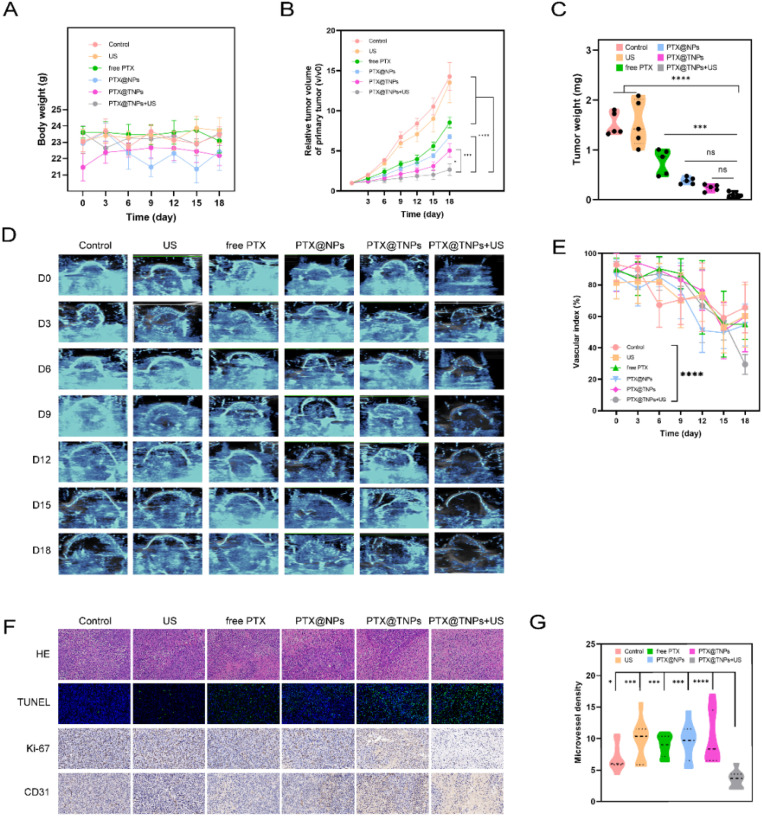
The treatment of mice in the control group, US group, free PTX group, PTX@NPs group, PTX@TNPs group, and PTX@TNPs + US group was analyzed. (A) Body weight of mice in each group; (B) relative tumor volume ratio of mice in each group; (C) the tumor weight of mice in each group; (D) and (E) superb microvascular imaging and quantitative analysis of VI in each group; (F) representative images of H & E staining, emmunohistochemical analysis of Ki-67 and CD31, and TUNEL assay of apoptosis in tumors. (G)The microvessel density of ultrasound combined with the targeted nanoparticles group was the lowest. ****P < 0.00001, ***P < 0.0001, **P < 0.001, *P < 0.05 and ns indicates *p* > 0.05.

Fig. 8A and B demonstrate the tumor growth inhibition observed across the six treatment groups. Notably, the PTX@TNPs + US group exhibited the strongest inhibitory effect, with a significantly lower tumor volume increase compared to the PTX@TNPs group (2.66 ± 1.72 *vs.* 5.04 ± 0.85, *P* = 0.0422, *n* = 5), PTX@NPs group (2.66 ± 1.72 *vs.* 6.77 ± 0.27, *P* = 0.0004, *n* = 5), and markedly lower than control, US, and free PTX groups (2.66 ± 1.72 *vs.* 14.28 ± 1.73, 13.53 ± 2.52, and 8.54 ± 0.65, respectively, *P* < 0.0001). These results confirm that both nanoparticle modification and ultrasound stimulation contribute to the enhanced therapeutic efficacy, with ultrasound offering additional benefit to PTX@TNPs treatment in TNBC.

Although final tumor weights (Fig. 8C) among the PTX@TNPs + US, PTX@TNPs, and PTX@NPs groups showed no statistically significant differences, this may be attributed to biological variability among animals, differences in necrosis or edema within tumors, or the presence of residual fibrotic tissue. Tumor volume better reflects treatment response during the experimental course, while endpoint tumor weight can be affected by post-treatment tissue composition. Nonetheless, both parameters point to the superior therapeutic outcome of the PTX@TNPs + US group.

Moreover, ultrasound also provides valuable insights for monitoring treatment efficacy. Superb microvascular imaging (SMI), a technique that evaluates tumor vascularity and microcirculation without contrast agents, was used to assess tumor response.^41,42^ As shown in Fig. 8D and E, the vascular index of the PTX@TNPs + US group was significantly reduced, correlating with decreased neovascularization (*P* < 0.0001, *n* = 15), further supporting the antiangiogenic and tumor-suppressive effects of the treatment.

### 3.8 Histological and immunohistochemical analysis

HE staining revealed histological changes in tumor cells across treatment groups. The control and US groups exhibited intact tumor structures with densely packed tumor cells, while the other four groups showed varying degrees of cell necrosis (Fig. 8F). Notably, the PTX@TNPs + US group displayed the most pronounced nuclear pyknosis and fragmentation.

TUNEL staining, used to detect apoptosis, showed minimal apoptotic cells in the control and US groups. In contrast, the other four treatment groups demonstrated increasing levels of apoptosis, with the PTX@TNPs + US group showing the most extensive apoptotic activity.

Ki-67 and CD31 immunohistochemical staining were applied to evaluate tumor cell proliferation and neovascularization. Ki-67 positive staining (brown nuclei) was abundant in the control and US groups but significantly reduced in the other treatment groups, especially in the PTX@TNPs + US group. For CD31 staining, although vascular staining appeared heterogeneous among sections, only the PTX@TNPs + US group exhibited a notable reduction in microvessel signal in most visual fields (Fig. 8F).

To quantitatively assess microvessel density (MVD), CD31-positive areas were analyzed using ImagePro software (Fig. 8G). The PTX@TNPs + US group showed the lowest MVD value, with statistically significant differences compared to other groups (*P* = 0.0379, *n* = 3). We note that variability in CD31 signal among sections may reflect intratumoral heterogeneity, yet the overall data confirm that PTX@TNPs + US treatment significantly inhibits tumor angiogenesis.

This study highlights the potential of the PTX@TNPs system in overcoming the limitations of conventional paclitaxel therapy by improving drug delivery efficiency and reducing systemic toxicity. The use of GE11 peptides for targeted delivery, combined with ultrasound-mediated drug release, represents a promising therapeutic strategy for TNBC. However, the development of drug resistance in TNBC, particularly against paclitaxel, remains a major challenge. Future research should focus on strategies to overcome paclitaxel resistance, potentially through combination therapy or by developing nanoparticles capable of delivering resistance-modulating compounds. Additionally, the long-term safety and efficacy of this nanocarrier system should be evaluated in clinical trials to fully realize its potential in TNBC treatment.

## 4 Conclusions

In summary, this study developed a targeted nanoparticle delivery system (PTX@TNPs) for paclitaxel (PTX), utilizing GE11 peptide-mediated targeting to enhance drug uptake in EGFR-overexpressing TNBC cells. Coupled with ultrasound, this strategy significantly improved the therapeutic efficacy of PTX by promoting tumor cell apoptosis, inhibiting growth, and reducing systemic toxicity. Our results highlight the potential of combining targeted drug delivery with ultrasound as a promising approach for improving chemotherapy outcomes in triple-negative breast cancer. Future studies should focus on overcoming challenges like drug resistance and optimizing treatment protocols.

## Ethical statement

All animal experiments were conducted in accordance with the Guidelines for the Care and Use of Laboratory Animals of Ningbo University, and were approved by the Animal Ethics Committee of Ningbo University (Approval No: NBU20220197).

## Author contributions

Zhenbin Xu: conceptualization, methodology, investigation, formal analysis, writing – original draft. Hongpeng Duan: data curation, validation, visualization. Yuling Shi: methodology, resources, investigation. Zixia Zhou: software, formal analysis, visualization. Zhuo Wei: data curation, validation. Xuechen Qian: investigation, resources. Jian Lu: project administration, supervision. Yuemingming Jiang: software, data curation. Feng Mao: resources, supervision. Nianyu Xue: formal analysis, writing – review & editing. Shengmin Zhang: conceptualization, funding acquisition, supervision, writing – review & editing.

## Conflicts of interest

There are no conflicts to declare.

## Data Availability

The authors declare that the data supporting the findings of this study are included within the article.
