# Correction: Ultrasound-mediated paclitaxel-loaded EGFR nanoparticles for targeted therapy in breast cancer

**DOI:** 10.1039/d5ra90090e

**Published:** 2025-08-12

**Authors:** Zhenbin Xu, Hongpeng Duan, Yuling Shi, Zixia Zhou, Zhuo Wei, Xuechen Qian, Jian Lu, Yuemingming Jiang, Feng Mao, Nianyu Xue, Shengmin Zhang

**Affiliations:** a Department of Ultrasonography, The First Affiliated Hospital of Ningbo University China fyyzhangshengmin@nbu.edu.cn; b Health Science Center, Ningbo University Ningbo China

## Abstract

Correction for ‘Ultrasound-mediated paclitaxel-loaded EGFR nanoparticles for targeted therapy in breast cancer’ by Zhenbin Xu *et al.*, *RSC Adv.*, 2025, **15**, 23136–23145, https://doi.org/10.1039/D5RA01016K.

The authors regret the omission of a funding acknowledgement in the original article. This acknowledgement is given here.

**Acknowledgements**

This research was critically supported by the following grants: Natural Science Foundation of Zhejiang Province (no. LBY23H180003) and Medical Science and Health Technology Project of Zhejiang Province (no. 2022KY319, 2022KY1065, and 2023KY1077).

The Royal Society of Chemistry apologises for these errors and any consequent inconvenience to authors and readers.

